# Time-course evaluation of blood glucose changes in response to insulin delivery in critically ill patients

**DOI:** 10.1186/cc13630

**Published:** 2014-03-17

**Authors:** F Bass, S Bird, N Hammond, J Myburgh, S Finfer

**Affiliations:** 1 Royal North Shore Hospital, St Leonards, NSW, Australia; 2 The George Institute for Global Health, Sydney, Australia

## Introduction

Intravenous insulin by infusion is commonly used for blood glucose control in the ICU and blood glucose is almost exclusively monitored by intermittent sampling. The rate of change in blood glucose concentration [BG] when the insulin infusion rate is changed is not known, and as a result the optimum time to measure [BG] after changing the infusion rate is unclear.

## Methods

Following institutional ethics approval and patient consent, using a GluCath Continuous Glucose Monitoring system sensor deployed via a radial artery catheter we studied the change in [BG] in response to a 1 unit/hour increase in the insulin infusion rate during the first 48 hours after cardiac surgery. [BG] was recorded every 10 seconds. Insulin was infused at a concentration of 1 unit/5 ml/hour via a volumetric pump. We recorded [BG] for 2 hours after changing the insulin infusion rate. Data affected by artifacts produced by blood draws and subsequent flushing of the arterial catheter were excluded and linear interpolation was used to estimate missing [BG] data.

## Results

**Figure 1:**
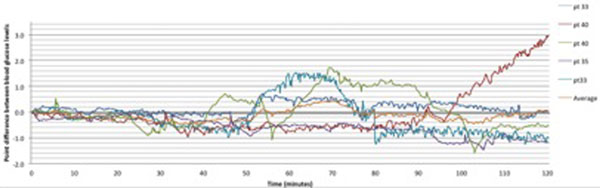
Blood glucose change after infusion change and after calibration.

There were five episodes where the insulin infusion increased by 1 unit/hour. [BG] decreased modestly for 50 minutes, after which there was marked interpatient variability in subsequent [BG] trend (Figure 1). The median (range) change in bG (mmol/l) at 30 minutes: -0.5 (-0.7/+0.1), 60 minutes: -0.1 (-0.7/+1.0), 90 minutes: -0.3 (-0.7/+0.6) and 120 minutes: -0.5(-1.2/+2.9).

## Conclusion

Within the first hour the change in [BG] was consistent but beyond this time it was highly variable. Further studies are needed to understand the dynamics of [BG] in response to changes in insulin infusion rates, but these data suggest [BG] should be checked hourly until stable.

